# The gut microbiota-short-chain fatty acid-inflammation axis in Parkinson's disease motor dysfunction: mechanisms and future of dietary interventions

**DOI:** 10.3389/fneur.2026.1889517

**Published:** 2026-09-10

**Authors:** Wanhong Wang, Wenyu Sun, Lihong Ma, Mengting Xu, Xu Dong

**Affiliations:** 1 School of Rehabilitation Medicine, Shandong University of Traditional Chinese Medicine, Jinan, Shandong, China; 2 Department of Rehabilitation and Physiotherapy, Affiliated Hospital of Shandong University of Traditional Chinese Medicine, Jinan, Shandong, China

**Keywords:** dietary intervention, gut microbiota, gut-brain axis, motor dysfunction, neuroinflammation, Parkinson's disease, short-chain fatty acids

## Abstract

Parkinson's disease (PD) is a neurodegenerative disorder characterized mainly by motor dysfunction that severely affects quality of life. In recent years, the gut microbiota and its metabolites, short-chain fatty acids (SCFAs), have become a focus in PD research. These factors are proposed to influence disease progression, potentially by modulating neuroinflammation and gut-brain axis signaling, although the causal relationship between specific microbial metabolites and clinical deterioration remains to be conclusively demonstrated in human studies. PD patients show characteristic gut dysbiosis, with fewer beneficial SCFA-producing bacteria and more pro-inflammatory bacteria, which may drive disease progression via the gut-brain axis. Dietary interventions can regulate the gut microbiota, increase SCFA levels, and reduce inflammation, and have shown promise for improving motor symptoms in PD. This review summarizes the current understanding of the “gut microbiota-SCFA-inflammation axis” in PD motor dysfunction, focusing on the potential mechanisms by which specific dietary strategies-prebiotics, probiotics, and the Mediterranean diet, and emerging patterns such as ketogenic diets (KD) and intermittent fasting (IF)-may ameliorate motor symptoms through modulation of this axis, while critically appraising the limitations and inconsistencies in the existing literature. By integrating disease-specific mechanistic insights with a translational evaluation of dietary interventions, this Review aims to complement existing immunological frameworks with a clinically oriented perspective on PD motor dysfunction. We also integrate recent preclinical and clinical evidence, discuss current research limitations, and offer perspectives on future therapeutic directions.

## 1 Introduction

Parkinson's disease (PD) is a common neurodegenerative disorder. Its pathology involves progressive loss of dopaminergic neurons in the substantia nigra and abnormal α-synuclein aggregation, leading to typical motor symptoms such as tremor, rigidity, and bradykinesia (1, 2). Current treatments mainly target the dopaminergic system with drugs like levodopa, but these only relieve symptoms and do not halt disease progression (2). Evidence has accumulated in recent years that gut microbiota dysbiosis may be an early event in PD, driving neuroinflammation and neurodegeneration through the gut-brain axis (3, 4). Gut microbiota ferment dietary fiber to produce short-chain fatty acids (SCFAs), including acetate, propionate, and butyrate. SCFAs are important energy sources for intestinal epithelial cells and also exert profound effects on the central nervous system by regulating immune homeostasis, suppressing inflammation, and maintaining intestinal barrier function (5, 6).

Studies have shown that fecal SCFA levels are significantly reduced in PD patients, while plasma acetate levels are abnormally elevated-a paradoxical finding possibly related to intestinal barrier disruption and systemic inflammation (7, 8). The reduction in SCFAs caused by gut dysbiosis further increases intestinal mucosal permeability, promoting the translocation of endotoxins such as lipopolysaccharide (LPS) into the bloodstream, activating microglia, and triggering neuroinflammation, which in turn accelerates dopaminergic neuron loss (9, 10). The “gut microbiota-SCFA-inflammation axis” serves as a core bridge connecting environmental factors (such as diet) to PD pathology (11). Its dysregulation is closely linked to motor symptoms and may also drive disease progression by affecting protein misfolding (e.g., α-synuclein aggregation), mitochondrial dysfunction, and autophagy-lysosomal pathway abnormalities (12). For example, butyrate, an important SCFA, exerts neuroprotective effects by inhibiting histone deacetylases (HDACs) and activating G protein-coupled receptors (e.g., GPR41/43), but its levels are markedly reduced in PD patients (10).

Based on the regulatory mechanisms of this axis, strategies such as dietary interventions (e.g., dietary fiber supplementation, probiotic/prebiotic use) and fecal microbiota transplantation (FMT) have shown potential disease-modifying effects (13, 14). For instance, in MPTP-induced PD mouse models, supplementation with butyrate-rich probiotics significantly improved motor dysfunction, reduced neuroinflammation, and protected dopaminergic neurons (15). Moreover, interventions targeting the gut microbiota may provide new avenues for early prevention and personalized treatment of PD by restoring immune-metabolic homeostasis of the gut-brain axis (16, 17). A deeper understanding of the “gut microbiota-SCFA-inflammation axis” in PD will not only help clarify the multifactorial pathogenesis of the disease but also lay the groundwork for developing safe and effective disease-modifying strategies, particularly dietary interventions (18, 19).

While comprehensive reviews have elucidated the fundamental immunomodulatory roles of SCFAs in health and disease, a focused synthesis of how SCFA dysregulation specifically contributes to motor dysfunction in PD-and how dietary strategies may counteract this process-remains lacking. This Review distinguishes itself from existing broad-scope immunological surveys by: (i) providing a disease-specific integration of the “gut microbiota-SCFAs-inflammation axis” within PD pathophysiology; (ii) critically appraising conflicting evidence, including the paradoxical fecal-plasma SCFA discrepancy and the limited efficacy on non-motor features; and (iii) offering a clinically oriented evaluation of dietary interventions as potential adjunctive therapies for motor symptom management. By bridging mechanistic understanding with translational application, this Review aims to complement rather than replicate existing general immunological frameworks.

## 2 Parkinson's disease and the gut microbiota-short-chain fatty acids-inflammation axis

### 2.1 Gut microbiota and motor function

Multiple metagenomic studies have consistently found significant compositional changes in the gut microbiota of PD patients, characterized by reduced α-diversity and overall dysbiosis (20). This dysbiosis includes increased relative abundance of pro-inflammatory bacteria and decreased abundance of beneficial bacteria with anti-inflammatory and SCFA-producing potential. Specifically, the abundance of pro-inflammatory bacteria belonging to Enterobacteriaceae is increased in PD patients (21). In contrast, SCFA-producing bacteria (especially butyrate producers) such as Faecalibacterium, Roseburia, and Bifidobacterium are significantly reduced (20, 22). A metagenomic study of Italian PD patients found that members of Lachnospiraceae, including Butyrivibrio, Pseudobutyrivibrio, Coprococcus, and Blautia, were decreased; these bacteria are associated with anti-inflammatory and neuroprotective effects (23). Another study also reported a decreasing trend of Prevotella in PD patients (24). Together, these compositional changes point to a pro-inflammatory state and impaired SCFA synthesis in the gut microenvironment of PD patients (25).

This specific dysbiosis pattern correlates with clinical severity. Gut microbiota composition is positively correlated with the severity of motor symptoms (e.g., Unified Parkinson's Disease Rating Scale Part III score, UPDRS-III) (26). For example, one study found a significant association between gut microbiota diversity and UPDRS-III scores (27). Dysbiosis is also associated with the rate of disease progression and the severity of specific motor symptoms such as postural instability and gait disturbance, suggesting that gut microbiota composition could serve as a biomarker and prognostic indicator for PD (26). Further research showed that the abundance of certain inflammation-related microbial genera (e.g., Enterobacteriaceae) increases with longer PD Hoehn and Yahr stages and disease duration, indicating that these bacteria may contribute to disease worsening (28). Functional analysis of the gut microbiota in PD supports this view, showing reduced carbohydrate fermentation and butyrate synthesis capacity, along with increased proteolytic fermentation and production of harmful amino acid metabolites (e.g., p-cresol and phenylacetylglutamine); these metabolic changes are strongly linked to constipation and intestinal dysfunction (25).

Growing evidence suggests that gut microbiota dysbiosis may occur before the onset of typical motor symptoms, already present in the prodromal phase. Similar gut microbiota alterations have been observed in patients with rapid eye movement sleep behavior disorder (RBD) (29), a known prodromal marker of PD. This supports the hypothesis that dysbiosis is a causative factor rather than merely a consequence of the disease (26). The “gut-origin” hypothesis proposes that changes in the intestinal environment (dysbiosis, barrier disruption, and inflammation) may promote α-synuclein misfolding and aggregation via pathways such as the vagus nerve, eventually ascending to the central nervous system and initiating neurodegeneration (30, 31). Therefore, understanding PD-specific gut microbiota changes will help reveal early disease pathology and provide new directions for microbiome-based early diagnosis and intervention (32).

It is important to acknowledge that while the majority of studies point toward a depletion of SCFA-producing taxa in PD, this pattern is not universally observed. Some cohorts have reported no significant differences in certain SCFA-producing genera, or even an increase in specific taxa depending on disease stage and medication status, particularly the use of catechol-O-methyl transferase (COMT) inhibitors or levodopa itself, which can confound microbial composition. Furthermore, geographical and dietary variations across study populations significantly impact baseline microbiota, potentially explaining inter-study discrepancies. Therefore, the causal relationship between specific microbial shifts and motor symptom severity requires cautious interpretation, as current evidence remains largely associative rather than causative in human settings.

### 2.2 Dysbiosis affects intestinal barrier function and systemic inflammation

Gut microbiota dysbiosis in PD patients is closely associated with impaired intestinal mucosal barrier integrity. Multiple studies have found significant downregulation of tight junction proteins (e.g., occludin and ZO-1) in the intestines of PD patients, leading to increased permeability-a “leaky gut” (33). This barrier disruption allows bacterial products such as LPS to translocate into the circulation, activating a systemic low-grade inflammatory response (34). For example, in a preeclampsia mouse model, gut dysbiosis led to decreased expression of ZO-1 and occludin, along with elevated serum levels of LPS and pro-inflammatory cytokines (TNF-α, IL-6), confirming a causal link between dysbiosis and leaky gut (34). Similarly, in an α-synuclein overexpressing (ASO) mouse model of PD, low-dose dextran sulfate sodium (DSS)-induced intestinal barrier dysfunction, while not directly worsening motor deficits, significantly increased intestinal permeability, suggesting that leaky gut may be an early event in PD pathology (33).

Translocation of bacterial products affects the central nervous system through multiple pathways. On one hand, pathogen-associated molecular patterns (PAMPs) such as LPS cross the damaged blood-brain barrier via the circulation; on the other hand, afferent vagal pathways transmit peripheral inflammatory signals to the brain. In an intestinal ischemia-reperfusion injury model, for instance, barrier disruption followed by bacterial translocation activated Toll-like receptor (TLR) and NF-κB pathways, triggering a systemic inflammatory response that subsequently disrupted the blood-brain barrier and exacerbated neuroinflammation (35). Clinical studies have also shown significantly elevated serum levels of inflammatory markers such as IL-1β, IL-6, and TNF-α in PD patients, which correlate positively with the severity of motor dysfunction (36). A study of fecal calprotectin in PD patients found that elevated levels were associated with intestinal inflammation and barrier injury, further supporting the existence of a “dysbiosis-leaky gut-inflammation-motor symptom” axis (36).

Gut dysbiosis may also exacerbate inflammation through reduced production of metabolites such as SCFAs. SCFAs (e.g., butyrate) not only provide energy for intestinal epithelial cells but also exert anti-inflammatory effects by inhibiting HDACs (37). In senescence-accelerated mice (SAMP8), decreased SCFA levels are associated with intestinal barrier damage and neuroinflammation, while supplementation with SCFA precursors restores tight junction protein expression and reduces inflammation. Similarly, in patients with liver cirrhosis, SCFA reduction due to dysbiosis is closely linked to intestinal barrier disruption and systemic inflammation, highlighting the central role of SCFAs in maintaining intestinal homeostasis (38). In summary, gut dysbiosis contributes to the progression of motor symptoms in PD through intestinal barrier disruption, systemic inflammation, and altered metabolite profiles, providing a theoretical basis for dietary interventions such as probiotic or SCFA supplementation (37, 38).

### 2.3 Short-chain fatty acids: key molecules of the gut-brain axis

SCFAs mainly include acetate, propionate, and butyrate, produced by gut microbiota through fermentation of dietary fiber (39). Among these, butyrate is particularly important for neuroprotection due to its strong anti-inflammatory activity and ability to inhibit HDACs (40). Butyrate regulates gene expression by inhibiting HDACs and directly modulates immune cell function by activating G protein-coupled receptors (e.g., GPR43), thereby reducing neuroinflammation (40). Propionate has also been shown to reduce inflammation in experimental arthritis by modulating synovial fibroblast function (40). These findings highlight the central role of SCFAs in immune regulation and neuroprotection.

While fecal levels of SCFAs, particularly butyrate, are consistently reported to be reduced in PD patients-aligning with the depletion of butyrate-producing bacteria-the profile of systemic (plasma/serum) SCFAs presents a more complex and paradoxical picture. Notably, Chen et al. (41) demonstrated that while fecal SCFAs were decreased, plasma acetate and propionate levels were significantly elevated in PD patients compared to controls, a finding corroborated by other studies. This discrepancy suggests a potential “leaky gut” phenomenon, where increased intestinal permeability allows for excessive translocation of bacterial metabolites into the bloodstream, or alternatively, reflects altered host energy metabolism and systemic inflammation. Consequently, relying solely on fecal SCFAs as a biomarker may be misleading, and the dynamic equilibrium between luminal production and systemic absorption must be considered when interpreting the role of SCFAs in PD pathophysiology.

SCFAs are not only energy substrates but also important signaling molecules. They exert extensive immunomodulatory and epigenetic effects by activating G protein-coupled receptors (e.g., GPR41, GPR43, GPR109A) and inhibiting HDACs (42). For example, the expression of GPR43 in cardiomyocytes and its regulation of cell function via the cAMP-PKA-CREB axis reveal the multiple functions of SCFAs in the cardiovascular and nervous systems (43). Additionally, SCFAs indirectly affect central nervous system health by regulating intestinal barrier function and reducing systemic inflammation (44). In PD, these SCFA functions may exert neuroprotective effects by inhibiting abnormal α-synuclein aggregation and alleviating neuroinflammation (5). Therefore, SCFAs, as key signaling molecules between the gut microbiota and the brain, offer new targets for PD treatment.

### 2.4 SCFA-mediated neuroinflammation

In the central nervous system, microglia are key targets of SCFAs. Multiple studies have shown that SCFAs such as butyrate regulate microglial phenotype polarization through dual mechanisms: HDAC inhibition and G protein-coupled receptor activation (45, 46). Specifically, butyrate upregulates anti-inflammatory gene expression by inhibiting HDAC activity and activates downstream signaling pathways via GPR43/41 receptors, promoting the conversion of microglia from a pro-inflammatory M1 phenotype to an anti-inflammatory, neuroprotective M2 phenotype (47). This phenotypic switch is accompanied by reduced release of pro-inflammatory factors (e.g., IL-1β, TNF-α) and increased neurotrophic factors (e.g., BDNF) (48). Animal experiments further confirm that supplementation with butyrate-producing bacteria (e.g., *Blautia producta*) significantly reduces the expression of iNOS and COX-2 in the substantia nigra while increasing the levels of M2 markers such as Arg-1 and YM-1 (45). Moreover, SCFAs can block excessive microglial activation by inhibiting the NF-κB and MAPK signaling pathways (46), thus playing a central role in regulating neuroinflammation.

Under PD pathology, SCFA deficiency due to gut dysbiosis significantly impairs the inhibition of microglial M1 polarization (49). Clinical studies have found that fecal butyrate levels in PD patients are negatively correlated with disease severity (45). This deficiency keeps microglia in a sustained pro-inflammatory state, releasing large amounts of reactive oxygen species (ROS) and inflammatory mediators (46). ROS directly damage mitochondrial function in nigral dopaminergic neurons (50) and also promote abnormal α-synuclein aggregation and fibrillization (47). Notably, SCFA deficiency also disrupts bidirectional gut-brain communication by reducing tight junction protein expression (e.g., ZO-1, occludin), increasing intestinal permeability, allowing pro-inflammatory substances such as LPS to enter the circulation, and further activating peripheral and central inflammatory responses (51). This vicious cycle ultimately leads to extensive loss of dopaminergic neurons and worsening motor dysfunction (45).

**Figure 1:**
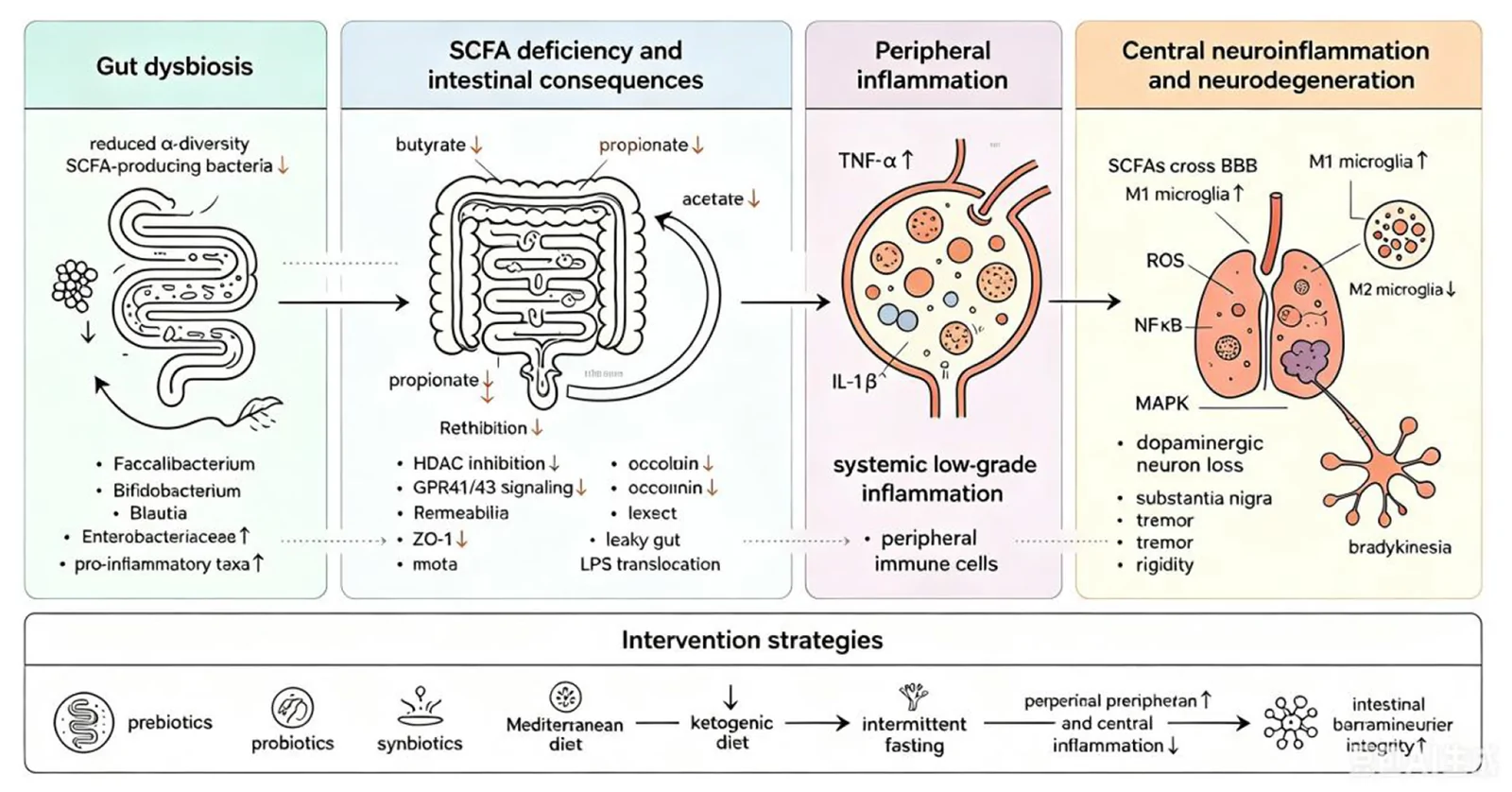
Schematic illustration of the “gut microbiota-SCFAs-inflammation axis” in Parkinson's disease motor dysfunction. This hand-drawn conceptual diagram depicts the multi-level mechanisms by which gut dysbiosis and SCFA deficiency contribute to neuroinflammation and motor impairment in PD, as well as the potential intervention points for dietary strategies.

Animal model studies provide direct evidence for the neuroprotective effects of SCFAs. In MPTP-induced PD mice, butyrate supplementation significantly reduced the activation of Iba-1+ microglia in the substantia nigra and increased the number of tyrosine hydroxylase (TH)-positive neurons (46). Similarly, oral administration of the butyrate-producing bacterium *Akkermansia muciniphila* reduced IL-6 and TNF-α levels in the hippocampus and striatum and improved rotational behavior and motor coordination in mice (52, 53). Mechanistically, these protective effects are related to inhibition of the RAS-NF-κB pathway regulated by SCFAs (45). Furthermore, fecal microbiota transplantation (FMT) experiments showed that PD models transplanted with young mouse microbiota (rich in SCFA-producing bacteria) exhibited more severe dopaminergic neuron damage than those transplanted with aged mouse microbiota (54), further confirming the critical role of SCFAs in PD pathology. These findings provide a solid experimental basis for treating PD by modulating SCFA levels through dietary or probiotic interventions. The key mechanisms described above are summarized visually in Figure 1.

## 3 Dietary effects on the gut microbiota-short-chain fatty acids-inflammation axis

### 3.1 Prebiotics and dietary

Fiber Prebiotics are non-digestible food components that selectively promote the growth and activity of beneficial gut bacteria, thereby improving host health (55). Based on their chemical structure and source, prebiotics can be classified into different subgroups; resistant oligosaccharides (e.g., fructooligosaccharides, galactooligosaccharides, and inulin) are the best-documented prebiotics in the literature (56). Dietary fiber, as an important source of prebiotics, has physicochemical properties (molecular size, monosaccharide composition, and glycosidic bond type) that determine its utilization by specific gut microbes, thus allowing precise shaping of the gut microbiota (57). In PD, gut dysbiosis is considered an important link in disease pathogenesis, and modulating the gut microecology through prebiotic intervention is emerging as a promising therapeutic strategy (26). Studies have shown that supplementation with specific prebiotics, such as fructooligosaccharides, inulin, and resistant starch, can significantly increase the abundance of beneficial bacteria such as Bifidobacterium and Lactobacillus in the gut of PD patients or model animals (58, 59). For example, a canine model study found that red ginseng-derived dietary fiber, as a prebiotic, increased gut microbiota diversity and enriched SCFA-producing microorganisms (60). These findings provide a theoretical basis for using prebiotics to target PD-associated gut microbiota.

By promoting fermentation by beneficial bacteria, prebiotic intervention effectively increases SCFA levels, particularly butyrate, in the gut and circulation (61). SCFAs are the main metabolites produced by gut microbial fermentation of dietary fiber and play key roles in maintaining intestinal barrier function, regulating immune responses, and suppressing inflammation (62). In PD, SCFA levels in the gut and feces are generally reduced, and this reduction correlates with disease severity (63). Prebiotic intervention increases SCFA production by increasing the abundance of SCFA-producing bacteria (e.g., Bifidobacterium, Lactobacillus, and certain Clostridiales) (64). For example, a pilot study in PD patients found that a dietary intervention rich in dietary fiber and lactulose significantly increased fecal SCFA content (64). *In vitro* fermentation experiments also confirmed that when PD patient fecal microbiota fermented prebiotics such as inulin, butyrate production significantly increased, although yields remained lower than in healthy controls (61). Increased SCFAs, especially butyrate, improve tight junctions between intestinal epithelial cells, enhance intestinal barrier function, and reduce systemic inflammatory markers (e.g., IL-1β, TNF-α) by inhibiting HDACs and activating G protein-coupled receptors (e.g., GPR43, GPR109A) (65, 66). A study in obese children showed that inulin supplementation did not show additional anti-inflammatory benefits (possibly due to Hawthorne effects), but intensive behavioral intervention itself reduced inflammatory cytokines, highlighting the importance of combining lifestyle and dietary interventions (67).

Preliminary clinical trials have shown that supplementation with specific prebiotics can improve constipation symptoms in PD patients, and some studies have observed slight improvements in motor symptom scores. Constipation is one of the most common non-motor symptoms of PD and is closely associated with gut dysbiosis (68). Prebiotics relieve constipation by increasing stool bulk, softening feces, and regulating intestinal motility (69). A study in hemodialysis patients (who often suffer from constipation) showed that supplementation with partially hydrolyzed guar gum (a prebiotic fiber) improved defecation status and increased fecal SCFA concentrations (69). In PD patients, an open-label, non-randomized study demonstrated that a 10-day prebiotic intervention was well tolerated and associated with beneficial changes in gut microbiota, SCFAs, and inflammatory markers (70). Another pilot study supplemented PD patients with 20 g of prebiotic fiber for 30 days and found altered gut microbiota and improved nutritional status indicators such as calf circumference (71). Furthermore, a study indicated that a fiber-rich Mediterranean diet, while increasing fiber intake, reduced fecal calprotectin (an intestinal inflammatory marker) levels in PD patients (72). Regarding motor symptoms, a randomized double-blind prospective study reported that supplementation with SCFAs (propionate and butyrate) and the prebiotic fiber 2′-fucosyllactose for 6 months led to clinically significant improvements in motor symptoms in PD patients and reduced levodopa dosage (73). However, these improvements vary across studies, and long-term effects and optimal dosages need to be confirmed by larger, more rigorously designed randomized controlled trials (RCTs) (74). Despite these encouraging findings regarding motor symptoms and constipation, it is crucial to temper expectations for broader clinical efficacy. Current randomized controlled trials (RCTs) are generally limited by small sample sizes, short intervention durations, and considerable heterogeneity in baseline microbiota. Moreover, the beneficial effects of these dietary interventions are predominantly observed in gastrointestinal and motor subscores, while their impact on debilitating non-motor features of PD-such as cognitive impairment, depression, and apathy-remains minimal or underexplored. Additionally, conflicting results exist, with some RCTs failing to show significant motor improvements over placebo. Therefore, while prebiotics offer a promising adjunctive therapy, they are unlikely to be a standalone disease-modifying solution, and their efficacy is highly contingent on individualized patient profiles. Overall, current evidence supports prebiotics as a potential adjunctive strategy for intervening in PD via the gut microbiota-SCFA-inflammation axis, but further research is needed to optimize intervention protocols and clarify clinical benefits.

### 3.2 Probiotics and synbiotics

Probiotic intervention directly corrects dysbiosis by supplementing live beneficial bacteria (e.g., Lactobacillus and Bifidobacterium strains). Multiple studies have shown that specific probiotic strains (e.g., *Lacticaseibacillus rhamnosus* GG and *Bifidobacterium lactis*) improve gut microecological balance by producing SCFAs, enhancing intestinal barrier function, antagonizing pathogens, and modulating immune responses (75, 76). For example, *L. rhamnosus* GG exhibited neuroprotective effects in a PD mouse model, with mechanisms related to upregulating glial cell line-derived neurotrophic factor (GDNF) expression in the striatum and increasing the abundance of specific bacteria (e.g., Clostridiales) (75). Moreover, probiotics can reduce neuroinflammation and oxidative stress by inhibiting pro-inflammatory cytokines (e.g., TNF-α, IL-6) and enhancing antioxidant enzyme activity (e.g., superoxide dismutase, glutathione peroxidase) (76). These effects collectively provide a theoretical basis for the potential therapeutic value of probiotics in PD motor dysfunction.

Clinical studies have shown that specific probiotic combinations significantly improve gastrointestinal symptoms (bloating, constipation) in PD patients and may positively affect UPDRS motor scores by reducing inflammatory factors (e.g., CRP). For example, a randomized controlled trial in PD patients found that a probiotic combination containing Lactobacillus and Bifidobacterium significantly relieved constipation symptoms while reducing serum CRP levels, which was associated with improved intestinal barrier function and reduced systemic inflammation (26, 68). Additionally, probiotic intervention can modulate gut microbiota structure, increasing the abundance of beneficial bacteria (e.g., Bifidobacterium and Lactobacillus) and reducing potentially pathogenic bacteria (e.g., Enterobacteriaceae), thereby indirectly affecting gut-brain axis signaling (77). Some studies have observed a trend toward improvement in UPDRS scores with probiotics, but results are inconsistent, possibly due to strain specificity, differences in treatment duration, or patient baseline microbiota characteristics (78). Crucially, the translational value of probiotics for holistic PD management warrants cautious interpretation, as their beneficial effects appear predominantly confined to gastrointestinal function and select motor subscores. In a randomized, double-blind, placebo-controlled trial specifically designed to evaluate probiotic supplementation in PD, Tan et al. (79) demonstrated that although constipation significantly improved (the primary endpoint), the intervention failed to elicit significant changes in non-motor features, including cognitive performance, depressive symptoms, and anxiety levels, compared to placebo. This discordance highlights the mechanistic complexity of the gut-brain axis, suggesting that peripheral microbial modulation via probiotics may have differential impacts on central motor vs. non-motor circuitries. Consequently, while probiotics represent a safe adjunctive therapy for managing PD-related constipation, their efficacy in alleviating the broader spectrum of non-motor symptoms remains unsubstantiated and necessitates further large-scale, multi-domain clinical investigations. Future long-term follow-up studies with larger sample sizes are needed to clarify the clinical benefits of probiotics for motor symptoms in PD.

Synbiotics (composite preparations of prebiotics and probiotics) theoretically synergize to promote probiotic colonization and function, representing an important direction for future dietary intervention research. However, high-quality clinical studies on synbiotics in PD are still limited. For example, one study combined the probiotic *L. rhamnosus* GG with the prebiotic polymannuronic acid (PM) and found that the neuroprotective effect was significantly superior to either component alone, with mechanisms involving SCFA-mediated anti-inflammatory/anti-apoptotic pathways and upregulation of BDNF expression (75). Another animal study showed that synbiotics (containing *Lactobacillus salivarius* AP-32 and its fermented supernatant) improved motor function in a 6-hydroxydopamine-induced PD model by directly enhancing host antioxidant enzyme activity and indirectly enriching commensal bacteria (e.g., SCFA-producing bacteria) (76). However, current synbiotic clinical trials in PD mostly focus on gastrointestinal endpoints, lacking systematic evaluation of motor symptoms (26, 68). Future research should optimize synbiotic formulations (e.g., selecting high-SCFA-producing strains combined with specific prebiotics) and design randomized controlled trials with motor function as the primary endpoint to verify their synergistic effects.

### 3.3 Whole dietary pattern interventions: the Mediterranean diet and emerging patterns

The Mediterranean diet, a typical healthy dietary pattern, is rich in whole grains, vegetables, fruits, legumes, nuts, olive oil, and fish. This dietary structure not only provides abundant dietary fiber and polyphenols but, more importantly, promotes the formation and maintenance of a healthy gut microbiota dominated by SCFA-producing bacteria (80). Studies have shown that the high fiber content of the Mediterranean diet provides ample fermentation substrates for gut microbes, promoting the proliferation of SCFA-producing bacteria such as Prevotella and Roseburia. Meanwhile, polyphenols in the diet, such as olive polyphenols and flavonoids, selectively promote the growth of beneficial bacteria, further optimizing gut microbiota composition. This approach to modulating gut microecological balance through dietary intervention offers new insights for the prevention and treatment of PD.

Multiple observational studies have confirmed that adherence to the Mediterranean diet is significantly associated with a reduced risk of PD and delayed disease progression (80). The mechanisms underlying this protective effect may involve the synergistic actions of various nutrients: first, SCFAs (e.g., butyrate, propionate) produced by fermentation of the abundant dietary fiber in the Mediterranean diet by gut microbiota regulate intestinal barrier function and reduce systemic inflammation; second, antioxidant components in the diet (e.g., vitamin E, polyphenols) neutralize free radicals and alleviate oxidative stress damage to dopaminergic neurons; third, ω-3 polyunsaturated fatty acids exert anti-neuroinflammatory effects by modulating microglial activity. These mechanisms collectively constitute the core links of the “gut microbiota-SCFA-inflammation axis,” providing a theoretical basis for the neuroprotective effects of the Mediterranean diet.

The unique advantage of the Mediterranean diet lies in its ability to provide a variety of bioactive substances with neuroprotective effects, including vitamins (e.g., B vitamins, vitamin D), minerals (e.g., magnesium, zinc), and polyphenolic compounds (80). These components support the homeostasis of the “gut microbiota-SCFA-inflammation axis” through different pathways: B vitamins act as coenzymes in neurotransmitter synthesis; polyphenols modulate gut microbiota composition and inhibit pro-inflammatory pathways such as NF-κB; ω-3 fatty acids promote the production of anti-inflammatory cytokines. Systematic reviews have shown that adherence to the Mediterranean diet significantly improves cognitive function and gastrointestinal symptoms in PD patients, which is closely associated with alterations in gut microbiota composition (particularly an increase in SCFA-producing bacteria) (80). As a comprehensive and sustainable dietary intervention strategy, the Mediterranean diet demonstrates unique value and application prospects in the management of PD through its multi-target regulatory mechanisms.

#### 3.3.1 Emerging dietary patterns: ketogenic diet, intermittent fasting, and plant-based approaches

Beyond the Mediterranean diet, other emerging dietary patterns have garnered attention for their potential to modulate the gut microbiota-SCFA axis in PD. The ketogenic diet (KD), characterized by high fat, adequate protein, and very low carbohydrate intake, has shown promise as a beneficial dietary intervention in PD. Notably, a study examining very low-calorie KD found an increase in the number of SCFA-producing bacteria, suggesting that ketogenic regimens may exert neuroprotective effects partly through microbiota modulation (81). However, classical KD formulations may unfavorably alter the gut microbiome and decrease SCFA levels, indicating that the effects of KD on SCFA production are complex and may depend on specific dietary composition and patient baseline characteristics (82). The emerging Mediterranean-ketogenic dietary interventions represent an attempt to harness the complementary benefits of both patterns.

Intermittent fasting (IF), an emerging dietary strategy alternating fasting and feeding cycles, has demonstrated neuroprotective effects in MPTP-induced PD mouse models through regulation of gut microbiota and SCFAs (83). Gas chromatography-mass spectrometry (GC-MS) analysis revealed that IF significantly increased fecal SCFA levels, while antibiotic intervention abolished the beneficial effects, confirming that gut microbiota mediated the neuroprotection of IF in PD. Beyond SCFAs, IF also modulates β-hydroxybutyrate (BHB) and brain-derived neurotrophic factor (BDNF), suggesting multi-modal mechanisms of action (84).

Plant-based diets, rich in dietary fiber and polyphenols, represent another promising approach. While direct evidence in PD populations remains limited, computational analyses have shown that plant-rich dietary patterns can remodel gut microbiota composition and enhance SCFA production (85). The predominantly plant-based low-protein, high-fiber diet is currently under investigation for its effects on the microbiome-immune-brain axis in PD patients (86).

Collectively, while the Mediterranean diet remains the most extensively studied dietary pattern in PD, emerging evidence supports the potential of ketogenic diets, intermittent fasting, and plant-based approaches as complementary or alternative strategies for modulating the gut microbiota-SCFA-inflammation axis. However, head-to-head comparisons and long-term clinical trials are urgently needed to establish optimal dietary recommendations for different PD subtypes.

## 4 Mechanisms of dietary intervention for motor dysfunction

### 4.1 Modulation of the gut-brain axis and vagus nerve function

A healthy gut microbiota maintains the function of enteroendocrine cells by producing SCFAs, thereby regulating the secretion of gastrointestinal peptides such as glucagon-like peptide-1 (GLP-1). These peptides can affect the function of the brainstem and basal ganglia via vagal afferent fibers or the circulatory system (87, 88). GLP-1 not only crosses the blood-brain barrier to act directly on the central nervous system but also regulates the activity of dopaminergic neurons in the nigrostriatal pathway through vagal afferent signals, thereby affecting motor function (89, 90). Moreover, SCFA reduction due to gut dysbiosis impairs the signaling capacity of enteroendocrine cells, which in turn affects vagal afferent signals, potentially contributing to the worsening of motor symptoms in PD (91, 92).

SCFAs (e.g., butyrate, propionate) can directly activate free fatty acid receptors (FFAR3) on vagal afferent terminals, transmitting signals to brain regions such as the nucleus tractus solitarius, thereby regulating neuroplasticity in the nigrostriatal pathway (93, 94). Epidemiological studies have found that vagotomy is associated with a reduced risk of PD, supporting the critical role of the vagus nerve in gut-brain communication (92, 95). Animal experiments further demonstrate that vagotomy blocks neuroinflammation and motor deficits induced by transplantation of depression-associated microbiota, confirming that the vagus nerve is an important pathway for microbiota-gut-brain signaling (88, 96). Additionally, vagus nerve stimulation (VNS) can improve motor function in PD model animals by increasing BDNF expression and inhibiting neuroinflammation (95, 97).

Dietary interventions, by optimizing gut microbiota composition and increasing SCFA production, may enhance protective bidirectional gut-brain communication. High-fiber diets promote the proliferation of specific bacteria (e.g., Prevotella, Roseburia), which produce SCFAs through fermentation of dietary fiber, thereby stabilizing dopaminergic system function (98, 99). For example, butyrate can activate vagal FFAR3 receptors, inhibit excessive microglial activation, reduce α-synuclein aggregation, and thus indirectly protect nigral dopaminergic neurons (100, 101). Clinical studies have also found that fecal SCFA levels in PD patients are negatively correlated with the severity of motor symptoms, suggesting that dietary modulation of the microbiota-SCFA-vagus nerve axis may be a new strategy for PD intervention (97, 102). Furthermore, probiotics (e.g., *Lactobacillus rhamnosus*) can increase splenic regulatory T cells via a vagus nerve-dependent pathway, inhibit hippocampal microglial activation, and improve neuroinflammation and motor coordination (103, 104).

### 4.2 Reduction of peripheral and central immune-inflammatory responses

Dietary interventions that elevate SCFA levels directly strengthen the intestinal barrier, reduce endotoxin translocation, and thereby inhibit the initiation of systemic inflammation. Gut dysbiosis is closely associated with impaired intestinal barrier function, allowing endotoxins such as LPS to translocate into the circulation, triggering systemic low-grade chronic inflammation (105). SCFAs, particularly butyrate, are key metabolites that maintain intestinal barrier integrity. Butyrate promotes the expression of tight junction proteins in intestinal epithelial cells and repairs damaged intestinal mucosa, effectively reducing the translocation of bacteria and their metabolites (e.g., LPS) (106). In intestinal inflammatory models such as inflammatory bowel disease, decreased SCFA levels produced by gut microbiota are directly associated with increased intestinal barrier permeability and exacerbated systemic inflammation (107). Dietary interventions (e.g., inulin supplementation) that restore gut microbiota balance and increase SCFA production have been shown to repair the intestinal barrier and reduce the release of pro-inflammatory cytokines, thereby inhibiting the initiation of systemic inflammation (108). This mechanism of controlling inflammation at the intestinal source is crucial for blocking subsequent inflammatory cascades to the central nervous system.

In the central nervous system, SCFAs (especially butyrate) can cross the blood-brain barrier, regulate microglial phenotype through HDAC inhibition, reduce the toxic effects of pro-inflammatory factors on neurons, and may promote the expression of neurotrophic factors. As key metabolites of gut microbiota, SCFAs can cross the blood-brain barrier and directly act on the central nervous system (109). Butyrate, as an HDAC inhibitor, regulates microglial phenotype and function. In *in vitro* models of neurodegenerative diseases such as Alzheimer's disease, SCFA mixtures (including butyrate) inhibit the secretion of pro-inflammatory cytokines (e.g., IL-1β, TNF-α) by microglia-like cells after immune stimulation and reduce the release of cytotoxic factors, thereby alleviating neuroinflammation (109). Additionally, SCFAs can regulate microglial phagocytic activity and oxidative stress responses by activating G protein-coupled receptors (109). In studies on multiple sclerosis, SCFAs such as butyrate and propionate have shown significant anti-inflammatory effects, influencing regulatory T cell expression and potentially improving the central immune environment by modulating blood-brain barrier permeability (110). These lines of evidence indicate that gut-derived SCFAs can directly exert immunomodulatory and neuroprotective effects in the central nervous system.

This systemic anti-inflammatory effect from the gut to the brain is one of the core mechanisms by which dietary interventions alleviate neurodegeneration and improve motor function. Biomarker studies show that effective dietary interventions are accompanied by decreased levels of inflammatory factors in serum and cerebrospinal fluid. The gut microbiota-SCFA axis constitutes a key bridge connecting peripheral and central immune inflammation. Dietary interventions reshape the gut microbiota, increase the abundance of protective bacteria (e.g., butyrate-producing Faecalibacterium), and elevate SCFA levels, thereby exerting anti-inflammatory effects at multiple levels (111). This systemic effect is first reflected peripherally, such as reducing serum levels of inflammatory markers like C-reactive protein and interleukin-6 (112). Subsequently, anti-inflammatory signals are transmitted to the brain via circulatory or neural pathways (e.g., the vagus nerve), modulating microglial activity and reducing neuroinflammation. In neurodegenerative diseases such as PD and Alzheimer's disease, patients often exhibit gut dysbiosis and altered SCFA levels, which are closely associated with central neuroinflammation and disease progression (113, 114). Preclinical studies have shown that SCFA supplementation (e.g., sodium propionate) reduces the expression of inflammatory factors in the central nervous system of Huntington's disease model mice (115). In human studies, following effective prebiotic or probiotic interventions, decreased levels of peripheral blood inflammatory factors have been observed and correlated with clinical symptom improvement (116). Therefore, monitoring changes in serum and cerebrospinal fluid inflammatory factor levels can serve as a potential biomarker for evaluating the neuroprotective effects of dietary interventions via the gut-brain axis.

### 4.3 Influence on energy metabolism and mitochondrial function

PD patients commonly exhibit mitochondrial dysfunction, manifested as impaired oxidative phosphorylation, accumulation of ROS, and dysregulated energy metabolism. SCFAs, especially butyrate, as the main metabolites of gut microbiota fermenting dietary fiber, not only inhibit HDACs but also serve as substrates for β-oxidation, directly participating in cellular energy metabolism regulation (117). Butyrate significantly enhances the antioxidant defense capacity of neurons and glial cells by promoting mitochondrial biogenesis and functional repair, thereby alleviating oxidative stress injury in PD pathology (118). Butyrate activates the peroxisome proliferator-activated receptor γ coactivator 1α (PGC-1α) pathway, enhances the activity of mitochondrial respiratory chain complexes, and upregulates the expression of uncoupling protein 2 (UCP2), reducing ROS production (119). Moreover, butyrate maintains mitochondrial network homeostasis by regulating mitochondrial dynamics (e.g., promoting phosphorylation of the fission protein Drp1), thereby improving neuronal energy supply (120).

Multiple studies have confirmed that the improvement of mitochondrial function by butyrate is cell-type specific. In neurons, butyrate inhibits apoptotic signaling pathways by increasing ATP production efficiency and reducing mitochondrial membrane potential depolarization (121). In astrocytes, butyrate upregulates the expression of monocarboxylate transporters (MCTs), promoting the transmembrane transport of lactate and ketone bodies, providing alternative energy substrates for neurons (122). Animal experiments further show that supplementation with butyrate precursors (e.g., inulin-type fructans) significantly increases butyrate levels in the brains of PD model mice, restores the morphology and function of mitochondria in dopaminergic neurons of the substantia nigra pars compacta, and improves motor coordination deficits (107). This protective effect is closely related to the activation of GPR41/GPR43 signaling pathways by butyrate, which inhibits NF-κB-mediated inflammatory responses and enhances mitophagy (123).

Dietary interventions provide indirect metabolic support to the brain by modulating gut microbiota composition or directly supplementing SCFA precursors. For example, a high dietary fiber diet can increase the abundance of butyrate-producing bacteria (e.g., Roseburia, Prevotella), thereby increasing colonic butyrate concentration (124). Butyrate then enters the systemic circulation via the portal vein or affects the central nervous system through vagal afferent signals. Preclinical studies have shown that a diet rich in resistant starch significantly improves gut dysbiosis in PD model animals, increases brain SCFA levels, and restores the activity of mitochondrial electron transport chain (ETC) complexes I and III (125). Furthermore, specific probiotics (e.g., Lactobacillus and Bifidobacterium) can improve host energy metabolism by producing SCFAs, with mechanisms involving activation of the AMP-activated protein kinase (AMPK) pathway, promoting fatty acid oxidation and glucose uptake (126). These findings provide a theoretical basis for intervening in PD motor dysfunction by targeting the gut microbiota-SCFA-mitochondria axis.

## 5 Future research directions and prospects

### 5.1 Novel therapeutic agents based on short-chain fatty acids or their precursors

SCFAs and their related targets have emerged as highly promising therapeutic directions for PD. Major strategies include: First, direct supplementation with SCFAs or their precursors. For example, butyrate has shown neuroprotective effects in animal models, but its oral bioavailability and blood-brain barrier permeability are low (46). To address this, researchers have developed colon-targeted delivery systems and prodrugs (e.g., tributyrin) to improve local concentrations and efficacy (61, 127). Second, the development of selective SCFA receptor agonists. Small molecule drugs targeting receptors such as GPR109A (e.g., MK-6892) can effectively inhibit neuroinflammation, alleviate motor symptoms, and exhibit good blood-brain barrier permeability (128, 129). The development of dual-target modulators may produce synergistic effects (130). Third, the use of synthetic biology to engineer probiotic strains. Modified strains can serve as “live biotherapeutics” to produce SCFAs (e.g., butyrate) in a targeted and sustained manner within the gut, offering a novel approach for treatment (49, 131).

### 5.2 Personalized dietary interventions

The core of constructing personalized dietary interventions for PD lies in the precise stratification of patients using multi-omics technologies. By integrating metagenomic, metabolomic, immunomic, and clinical data, a “microbiota-metabolite-immunity” classification system can be established to identify PD subtypes with distinct gut microecology, SCFA metabolic profiles, and inflammatory characteristics, laying the foundation for precision nutrition interventions (132, 133). Artificial intelligence and machine learning are key tools for developing personalized regimens. They can analyze complex associations within multi-omics data and intelligently recommend optimal dietary combinations for different subtypes. For example, for subtypes deficient in butyrate-producing bacteria, a diet rich in specific dietary fibers may be recommended to promote the growth of beneficial bacteria; for highly inflammatory subtypes, increasing the intake of anti-inflammatory nutrients may be advised (134–136). To validate and optimize such highly personalized strategies, innovative trial designs such as N-of-1 or micro-randomized trials are needed. These methods can dynamically evaluate intervention effects at the individual level and adjust regimens based on real-time monitoring of microbiota, metabolites, and symptom data, thereby maximizing therapeutic outcomes and providing new insights into the heterogeneity of PD (137, 138). Ultimately, this multi-omics-based precision nutrition platform is expected to become an important component of comprehensive PD management.

### 5.3 Multimodal therapeutic strategies

The management of PD is trending toward multimodal strategies integrating diet, pharmacotherapy, and non-pharmacological interventions. Dietary interventions can influence the efficacy of core medications by modulating the “gut microbiota-SCFA-inflammation axis.” For example, high-fiber or Mediterranean diets may optimize the absorption and metabolism of levodopa by improving the intestinal environment and microbiota composition. At the same time, dietary modulation can synergize with neuroprotective mechanisms. SCFAs (e.g., butyrate) and polyphenols may enhance the effects of neuroprotective drugs or directly exert beneficial actions through anti-inflammatory and epigenetic regulatory pathways. Furthermore, combining personalized diet with non-pharmacological therapies such as exercise rehabilitation and cognitive training may produce synergistic effects. Evidence suggests that targeted nutritional support (e.g., specific prebiotic supplementation before or after exercise) may further enhance motor and cognitive improvements through mechanisms such as the “gut-brain-muscle axis.” Future research should focus on exploring the precise coordination and timing of these interventions, developing individualized integrated plans based on patients' microbiota and clinical characteristics, and thereby establishing evidence-based comprehensive management pathways for PD.

## 6 Conclusion and limitations

The conceptual framework of the “gut microbiota-short-chain fatty acid-inflammation axis” offers a valuable heuristic model for understanding the potential mechanisms underlying motor dysfunction in PD. Current evidence suggests a plausible link whereby reduced production of microbial metabolites (e.g., butyrate, propionate) may contribute to peripheral immune activation and alterations in blood-brain barrier permeability, potentially fostering a neuroinflammatory environment that could promote the selective loss of dopaminergic neurons in the substantia nigra. This framework helps contextualize the observed clinical association between gut dysbiosis and worsening motor symptoms, encouraging a shift from a purely “brain-centered” view toward a systems-biology perspective of “gut-brain bidirectional regulation. However, it is critical to emphasize that the majority of human data currently support correlation rather than causation; definitive proof of a unidirectional, disease-driving axis awaits rigorous longitudinal and interventional studies. Nonetheless, this paradigm highlights promising avenues for future mechanistic exploration and potential therapeutic modulation.

However, existing studies still exhibit differences and controversies regarding specific mechanisms and intervention effects. For example, subjective biases in dietary recording need to be overcome by standardized metabolic cage techniques; there is inconsistency between central SCFA concentrations and fecal levels; and the long-term safety of high-fiber diets (e.g., bloating) requires careful consideration. Above all, while the gut microbiota-SCFA-inflammation axis represents an exciting frontier in PD research, translating these preclinical and observational findings into clinical practice will require a fundamental shift in interpretation—from viewing these associations as deterministic pathways to recognizing them as modifiable risk factors that operate within a complex, multifactorial disease network.
